# The modifier effect of physical activity, body mass index, and age on the association of metformin and chronic back pain: A cross-sectional analysis of 21,899 participants from the UK Biobank

**DOI:** 10.1371/journal.pone.0282205

**Published:** 2023-02-28

**Authors:** Ana Paula Carvalho-e-Silva, Paulo H. Ferreira, Alison R. Harmer, Jan Hartvigsen, Manuela L. Ferreira

**Affiliations:** 1 Faculty of Medicine and Health, Musculoskeletal Pain Hub, Charles Perkins Centre, The University of Sydney, Sydney, Australia; 2 John Walsh Centre for Rehabilitation Research, Sydney Medical School Northern, University of Sydney, Sydney, Australia; 3 Sydney Musculoskeletal Health, Research Centre, University of Sydney, Sydney, Australia; 4 Sydney School of Health Sciences, Faculty of Medicine and Health, The University of Sydney, Sydney, Australia; 5 Department of Sports Science and Clinical Biomechanics, Center for Muscle and Joint Health, University of Southern Denmark, Odense, Denmark; 6 Chiropractic Knowledge Hub, Odense, Denmark; 7 Sydney Musculoskeletal Health, The Kolling Institute, School of Health Sciences, Faculty of Medicine and Health, The University of Sydney, Sydney, Australia; Fondazione Toscana Gabriele Monasterio, ITALY

## Abstract

**Background:**

There is growing evidence of the anti-inflammatory effect of the anti-diabetic drug metformin and its use to reduce pain. However, we currently lack studies investigating whether metformin is associated with a reduction in chronic back pain prevalence when considering physical activity levels, body mass index (BMI), and age.

**Objective:**

To investigate whether use of metformin is associated with lower levels of reporting of chronic back pain in a large cohort with type 2 diabetes when stratified for physical activity, BMI, and age.

**Methods:**

This is a cross-sectional study of 21,889 participants with type 2 diabetes who were drawn from the UK Biobank database. We investigated whether people using metformin reported a higher prevalence of chronic low back pain than those who did not. Type 2 diabetes, chronic back pain, and metformin were self-reported. Participants were stratified according to their physical activity level (low, moderate and high), BMI (normal, overweight, and obese), and age (40 to <50; 50 to < 60; and ≥60 years). Logistic regression models were built for each physical activity level, BMI and age category to investigate the prevalence of chronic back pain amongst those using and not using metformin.

**Results:**

Participants who were using metformin and who had low levels of physical activity [OR 0.87, 95%CI 0.78 to 0.96] or who were obese [OR 0.90, 95%CI 0.86 to 0.98] or older [OR 0.85, 95%CI 0.78 to 0.93] had lower odds of reporting chronic back pain than their counterparts.

**Conclusion:**

The anti-diabetic drug metformin might reduce prevalence of chronic low back pain in people who are older, overweight, or less active. These findings should be confirmed in studies using a longitudinal design.

## Introduction

Back pain is the most prevalent type of musculoskeletal pain and is the leading cause of years lived with disability globally. Diabetes is a common comorbidity amongst those with back pain, for example, people with back pain have 35% greater odds [odds ratio (OR) 1.35;95%CI 1.20–1.52] of having type 2 diabetes mellitus than those without back pain, [1] and the co-occurrence of the conditions results in higher levels of pain, reduced quality of life, and poorer physical function when compared to either condition occurring in isolation. [2, 3]

One first-line medication commonly prescribed for type 2 diabetes is metformin. [4] Metformin acts by reducing hepatic glucose production, increasing peripheral glucose uptake, and hence improving glycaemia. [5] However, metformin has also been suggested to have pleiotropic effects. [6, 7] For example, metformin reduces the incidence of cardiovascular events by 17% [hazard risk (HR) 0.83;95%CI 0.78–0.89] in people with type 2 diabetes. [8] Another possible pleiotropic effect of metformin is pain modulation. [9–11] People with type 2 diabetes who have been prescribed metformin report lower levels of lumbar radiculopathy pain [mean of -1.85 points (0–10 pain scale)] than those not taking metformin. [9] In a cohort of 21,899 people with type 2 diabetes from the UK Biobank, we demonstrated [12] that those who took metformin had 13% lower odds [OR 0.87;95%CI 0.81–0.93] of reporting back pain compared to those not taking metformin. In contrast, previous research has failed to identify any effects of metformin in knee pain severity between people with diabetes using or not metformin over 4 years (p = 0.54); [13] as well as in the use of metformin to reduce body pain in a smaller retrospective study with a cohort with diabetes. [9]

Metformin activates adenosine monophosphate-activated protein kinase (AMPK) activation and represses chronic low-grade inflammation [14] with the AMPK pathway proposed as the mechanism through which metformin acts on pain. [11] AMPK activation suppresses the mammalian target of rapamycin (mTOR) pathway, [11] which in turn results in decreased sensitisation and nociceptor excitability of the peripheral nervous system in people with chronic pain. [15–17] In mice, the use of metformin has been shown to decrease postsurgical pain. [18] AMPK activation is also influenced by exercise, obesity, age, and sex. Skeletal muscle AMPK is activated by exercise. [19, 20] In contrast, AMPK activity is 45% lower in the visceral, rather than subcutaneous adipose tissue, of people with obesity and insulin resistance [21] and 14% lower in the muscles of older people and women, compared to younger and male counterparts. [22]

Only a few previous studies, all with small sample sizes, have examined the relationship between musculoskeletal pain and metformin; and the modifying effects of physical activity, BMI, and age on this relationship are not known. Using a very large population-based sample of people with type 2 diabetes, this study aimed to investigate the role of physical activity, BMI, and age on the relationship between use of metformin and the prevalence of chronic back pain.

## Methods

This study used cross-sectional data from participants with type 2 diabetes who attended the baseline assessment for the UK Biobank study in the 2006–2010 period. Participants completed a self-report questionnaire at baseline that included questions on lifestyle, family history, and health conditions such as musculoskeletal pain and diabetes. [23] Participants were subsequently interviewed by a nurse to validate the self-reported data, including data on medication intake and prevalence of health conditions. The UK Biobank: Protocol for a large-scale prospective epidemiological resource can be found elsewhere. [24]

All participants provided consent, and ethics approval was provided by the NHS National Research Ethics Service (Ref: 16/NW/0274).

In the UK Biobank study, approximately 500,000 adult participants were assessed at baseline (2006–2010), with 26,395 participants reporting having diabetes. Our study sample comprised 21,889 people with type 2 diabetes.

In this study, we included only people classified as having type 2 and “generic” diabetes and responded to the chronic low back pain question (yes or no). We excluded people with type 1 and gestational diabetes and those who took insulin during their first year of diagnosis. Participants who did not know or preferred not to answer the diabetes and chronic low back pain question were also excluded.

Those 21,889 were categorised into three categories for each modifier: Body Mass Index (BMI <18.5 kg/m^2^, underweight; BMI 18.5–24.9 kg/m^2^, normal weight; BMI 25–29.9 kg/m^2^, overweight; BMI ≥30 kg/m2, obese); Age (40 to <50; 50 to < 60; and ≥60 years old)) and Physical Activity Level according to IPAQ algorithms (low, moderate, or high levels of physical activity). For each modifier, the relationship between metformin users and the prevalence of chronic back pain was investigated.

### Type 2 diabetes (study sample)

Type 2 diabetes was self-reported. Participants who reported “Yes” to the following question at the baseline self-reported questionnaire were included: “Has a doctor ever told you that you have diabetes?”. The type of diabetes (type 1, type 2, gestational and “generic”) was determined during the interview with the nurse. A total of 21,889 cases of type 2 diabetes were identified and included in the analyses.

### Metformin (exposure)

During the verbal interview with the study nurse at the baseline, the participant’s medical history and regular prescription medication, such as metformin, were determined. Participants who reported diabetes were asked about taking regular prescribed medication. If they confirmed regularly taking medication, the following question was asked: Can you now tell me what these are? Then, patients listed their medication/s for the specific condition. For our study purpose, participants with type 2 diabetes were dichotomised into those who reported taking or not taking metformin to manage their condition. Therefore, we defined people who had type 2 diabetes who reported using metformin medication as the metformin group and those participants who reported having diabetes but not taking metformin as the group not using metformin.

### Chronic back pain (outcome)

Chronic back pain was assessed using a self-reported questionnaire, in which participants were first asked whether they had experienced pain that had interfered with their usual activities in the last month. The following options were given to the participants: none, prefer not to answer, pain all over the body, facial pain, one or more of the musculoskeletal pain sites–neck or shoulder pain, back pain, hip pain, knee pain”. If participants provided an affirmative answer for back pain, a further question was asked about whether the participant had experienced this pain for more than three months. Those participants reporting back pain for more than 3 months were classified as having chronic back pain. Participants not reporting back pain as a pain site in the first question and/or not reporting back pain for more than 3 months were classified as not having chronic back pain. Participants who responded “prefer not to answer” or “pain all over the body” were excluded from our study.

### Physical activity, BMI, and age (modifiers)

Physical activity was assessed using the International Physical Activity Questionnaire (IPAQ) short form, which was included with the self-report questionnaire. This a validated instrument with reliability and intraclass correlation coefficients between 0.65 and 0.57 [25, 26]. Participants reported the number of hours and days spent walking or participating in moderate or vigorous physical activities, per week. We used the IPAQ algorithm and protocol to categorise participants as having low, moderate, or high levels of physical activity. BMI was calculated using height and weight measured at baseline assessment. Participants were initially categorised into four BMI groups following the World Health Organization cut-points: underweight (BMI <18.5 kg/m^2^), normal weight (BMI 18.5–24.9 kg/m^2^), overweight (BMI 25–29.9 kg/m^2^), and obese (BMI ≥ 30.0 kg/m^2^). [27] Only five participants were categorised as underweight; hence we grouped them with the normal weight category and only used three BMI categories for the analyses. The UK Biobank study only recruited participants aged 40 years or older, therefore, participants were stratified according to the following age groups: 40 to <50; 50 to < 60; and ≥60 years old.

### Assessment of covariates

Education attainment was dichotomised as more and less extensive education. In the first group, we combined those who had achieved university or college degrees, higher secondary education, vocational qualifications, or other professional qualifications. In the second group, we combined those who attained secondary school education or the equivalent or none of the above educational degrees. Smoking status was self-reported and categorised as frequent/occasional smoker or non-current/ex-smoker. Cardiovascular disease and mental health categories were created by grouping appropriate comorbidities from the self-reported list (n = 476) provided by the UK Biobank. Age, BMI, and physical activity were also considered potential confounders in the analytical models where they were not considered the primary effect modifier.

### Statistical analysis

We summarised the demographic data, the prevalence of chronic back pain, physical activity level, BMI, and age according to those using or not using metformin (Table 1). Associations between metformin and chronic back pain were stratified by BMI (normal, overweight, obese), engagement in physical activity (low, moderate, high), and age (40 to <50; 50 to < 60 and ≥60 years). These associations were examined for the whole cohort, as well as when stratified by gender, using logistic regression models. We also analysed the interaction between metformin and modifiers (age, physical activity, and BMI) as a continuous variable in the logistic regression to investigate the association between metformin and chronic low back pain for the whole cohort as well as for females and males only. The covariates in the multivariate analyses for the models—physical activity, BMI and age—were retained when the association between the covariate and the outcome (chronic back pain) was less than 0.2 (p-value) in univariate models. [28]. Statistical significance was set at p<0.05, and data analyses were conducted using *v*ersion 16 (StataCorp. 2017. Stata Statistical Software: Release 16. College Station, TX: StataCorp LLC).

**Table 1:** Anthropometric data, the prevalence of metformin use, back pain status, BMI, and physical activity levels among people with type 2 diabetes.

Variables	Using metformin ^#^		Not using metformin ^#^		
	Mean (SD) or%	n	Mean (SD) or%	n	P value
Age (yr)	60.0 (6.8)	12,699	60.7 (6.6)	9,190	0.09
Duration of diabetes (yr)	8.0 (8.6)	12,312	6.4 (8.8)	8,873	0.12
Chronic Back pain^a^	23.3	2,810	24.5	2,145	0.04
Female	36.2	4,595	36.7	3,369	0.41
Male	63.8	8,104	63.3	5,821	0.42
Smokers^d^	10.9	1,363	10.6	968	0.56
Body mass index (kg/m^2^)^b^	32.1 (5.8)	12,576	31.2 (5.6)	9,104	0.90
Body mass index categories					
Normal weight	7.7	977	11.0	1,006	0.01
Overweight	32.5	4,084	35.8	3,258	0.01
Obese	59.8	7,515	53.2	4,840	0.01
Physical activity level ^c^					
Low	43.7	5,544	41.3	3,791	0.01
Moderate	35.2	4,474	35.7	3,284	0.44
High	21.1	2,681	23.0	2,115	0.01
Pain medication	5.0	628	5.2	485	0.27

SD: standard deviation; n: number of participants

^a^ Prevalence (%)

^**#**^ Percentage within the group

^b^ Body mass index classified according to WHO

^c^ classification according to short IPAQ scoring.

^d^ Smoker, participants who reported being frequent or occasional smokers

## Results

Data from 21,889 people with type 2 diabetes mellitus were used in this study (Table 1).

Table 2 shows the results of the regression models for the association between metformin and the reporting of chronic back pain according to low, moderate, and high levels of physical activity level. Use of metformin was associated with a reduction in the odds of reporting chronic back pain across lower levels of physical activity when adjusted for BMI, educational level, smoking status and reporting cardiovascular or mental health comorbidities [adjusted OR 0.87;95%CI 0.78–0.96] in comparison to those not using metformin. When results were stratified by gender, similar results were observed for women but not men.

**Table 2:** Association between metformin and back pain considering different levels of physical activity engagement.

Physical activity level									
	Low			Moderate			High		
	OR (95% CI)	p value	n	OR (95% CI)	p value	n	OR (95% CI)	p value	n
**Whole cohort**									
Cases/no-cases	2,454/6,308			1,567/5,914			934/3,609		
Unadjusted	0.91 (0.83–1.00**)**	0.06	8,762	0.94 (0.84–1.05)	0.29	7,481	0.92 (0.80–1.06)	0.27	4,543
Adjusted^a^	**0.87 (0.78–0.96)**	0.01	8,385	0.89 (0.79–1.01)	0.17	7,271	0.90 (0.77–1.04)	0.17	4,304
**Females**									
Cases/no-cases	1,089/2,192			599/1,943			377/1,208		
Unadjusted	**0.86 (0.74–0.99)**	0.04	3,281	0.94 (0.78–1.13)	0.56	2,542	1.02 (0.81–1.29)	0.84	1,585
Adjusted^a^	**0.84 (0.72–0.98)**	0.03	3,140	0.91 (0.75–1.10)	0.35	2,474	0.96 (0.75–1.22)	0.75	1,524
**Males**									
Cases/no-cases	1,365/4,116			968/3,971			557/2,401		
Unadjusted	0.95 (0.84–1.08)	0.46	5,481	0.93 (0.81–1.07)	0.37	4,939	0.87 (0.72–1.05)	0.15	2,958
Adjusted^a^	0.89 (0.78–1.02)	0.10	5,245	0.89 (0.76–1.02)	0.11	4,804	0.86 (0.71–1.05)	0.16	2,780

CI: confidence interval, OR: Odds ratio, Case/no-cases of chronic back pain

^a^ Adjusted for BMI (continuous), education, smoking status, cardiovascular and mental health conditions.

Statistical significance: p<0.05

Table 3 shows the results of the logistic regression models for the associations between metformin and chronic back pain by BMI categories. The use of metformin, compared to those not taking metformin, was associated with lower odds of reporting chronic back pain only in obese participants [adjusted OR 0.90; 95%CI 0.86–0.98]. When results were stratified by gender, no significant associations were found among BMI categories.

**Table 3:** Association between metformin and back pain considering body mass index classification.

Body Mass Index									
	Normal			Overweight			Obese		
	OR (95% CI)	p value	n	OR (95% CI)	p value	n	OR (95% CI)	p value	n
**Whole cohort**									
Cases/no-cases	310/1,589			1,399/5,650			3,196/8,458		
Unadjusted	0.83 (0.65–1.06)	0.14	1,899	0.90 (0.80–1.01)	0.08	7,049	**0.91 (0.84–0.99)**	**0.03**	11,654
Adjusted	0.83 (0.65–1.07)	0.18	1,831	0.89 (0.79–1.01)	0.07	6,808	**0.90 (0.86–0.98)**	**0.02**	11,321
**Females**									
Cases/no-cases	109/582			472/1,573			1,468/3,149		
Unadjusted	0.97 (0.64–1.47)	0.91	691	0.88 (0.71–1.08)	0.23	2,045	0.90 (0.80–1.02)	0.12	4,617
Adjusted	0.97 (0.65–1.48)	0.91	673	0.87 (0.70–1.08)	0.22	1,978	0.90 (0.80–1.03)	0.13	4,480
**Males**									
Cases/no-cases	201/1,007			927/4,077			1728/5,309		
Unadjusted	0.76 (0.56–1.03)	0.08	1,208	0.90 (0.78–1.04)	0.19	5,004	0.93 (0.83–1.04)	0.21	7,037
Adjusted	0.76 (0.56–1.05)	0.10	1,158	0.90 (0.78–1.04)	0.17	4,830	0.91 (0.81–1.02)	0.12	6,841

CI: confidence interval, OR: Odds ratio, Case/no-cases of chronic back pain

^a^ Adjusted for education, smoking status, cardiovascular and mental health conditions.

Statistical significance: p<0.05

Table 4 shows the regression model results for the association between metformin and chronic pain in the age groups 40 to <50 years, 50 to < 60, and≥60 years. Metformin use, compared to those not using metformin, was statistically associated with decreased odds of reporting chronic back pain among those in the oldest age group (≥60 years) [adjusted OR 0.85; 95%CI 0.78–0.93]; similar results were observed for males [adjusted OR 0.83;95%CI 0.72–0.95] and females [adjusted OR 0.87;95%CI 0.78–0.97]. No associations were observed for people in the younger age groups.

**Table 4:** Association between metformin and back pain considering age classification.

Age									
	40–50 years			50–60 years			60–72 years		
	OR (95% CI)	p value	n	OR (95% CI)	p value	n	OR (95% CI)	p value	n
**Whole cohort**									
Cases/no-cases	470/1,636			1,633/4,956			2,852/9,239		
Unadjusted	0.97 (0.78–1.20)	0.78	2,106	0.98 (0.88–1.10)	0.85	6,589	**0.90 (0.82–0.98)**	**0.01**	1,2091
Adjusted	0.92 (0.74–1.14)	0.45	2,022	0.94 (0.83–1.06)	0.32	6,345	**0.85 (0.78–0.93)**	**0.01**	1,1593
**Females**									
Cases/no-cases	214/547			716/1,714			1,135/5,343		
Unadjusted	1.00 (0.72–1.38)	0. 97	761	1.07 (0.90–1.28)	0.41	2,430	**0.83 (0.73–0.96)**	**0.01**	4,217
Adjusted	0.88 (0.62–1.24)	0.47	728	1.02 (0.85–1.23)	0.78	2,345	**0.83 (0.72–0.95)**	**0.01**	4,058
**Males**									
Cases/no-cases	256/1,089			917/3,242			1,717/6,157		
Unadjusted	0.98 (0.74–1.31)	0.93	1,345	0.94 (0.81–1.09)	0.43	4,159	0.93 (0.84–1.04)	0.24	7,874
Adjusted	0.97 (0.72–1.31)	0.85	1,294	0.90 (0.77–1.08)	0.21	4,000	**0.87 (0.78–0.97)**	**0.02**	7,535

CI: confidence interval, OR: Odds ratio, Case/no-cases of chronic back pain

^a^ Adjusted for BMI (continuous), education, smoking status, cardiovascular and mental health conditions.

Statistical significance: p<0.05

Table 5 shows the regression model results for the association between metformin and chronic pain when the interaction between metformin physical activity, BMI and age were performed, respectively. These interaction analyses were not significant, and no multiplicative effects were observed.

**Table 5:** Association between metformin and back pain considering the interaction between metformin and physical activity levels, BMI and age.

Physical activity									
	Whole cohort			Female			Male		
	OR (95% CI)	p value	n	OR (95% CI)	p value	n	OR (95% CI)	p value	n
Metformin	0.89 (0.81–0.98)	0.02	14,906	0.84 (0.72–0.99)	0.04	4,952	0.92 (0.82–1.04)	0.23	9,954
Physical activity^a^	0.99 (0.99–1.00)	0.26		0.99 (0.99–1.00)	0.39		0.99 (0.99–1.00)	0.60	
Metformin x Physical activity	0.99 (0.99–1.00)	0.85		0.99 (0.99–1.00)	0.57		0.99 (0.99–1.00)	1.00	
**BMI**									
Metformin	0.93 (0.64–1.34)	0.70	19,960	0.93 (0.53–1.62)	0.80	7,131	0.88 (0.52–1.48)	0.63	12,829
BMI^b^	1.04 (1.03–1.05)	<0.01		1.04 (1.03–1.06)	<0.01		1.03 (1.02–1.05)	0.00	
Metformin x BMI	0.99 (0.98–1.00)	0.805		0.99 (0.98–1.01)	0.89		0.99 (0.99–1.00)	0.96	
**Age**									
Metformin	1.09 (0.60–2.00)	0.78	20,136	1.09 (0.42–2.86)	0.84	7,186	1.18(0.53–2.62)	0.67	12,950
Age^b^	1.01(0.99–1.00)	0.71		0.99 (0.98–1.01)	0.57		1.00 (0.99–1.01)	0.21	
Metformin X Age	0.99 (0.98–1.01)	0.59		0.98 (0.98–1.01)	0.72		0.99 (0.98–1.00)	0.52	

CI: confidence interval, OR: Odds ratio

^a^ Physical activity as a continuous variable (METs)

^b^ BMI and age as continuous variables; Statistical significance: p<0.05

Physical activity analysis adjusted for BMI education, smoking status, cardiovascular and mental health conditions.

BMI analysis adjusted for education, smoking status, cardiovascular and mental health conditions.

Age analysis adjusted for BMI, education, smoking status, cardiovascular and mental health conditions.

## Discussion

Our findings suggest that the use of the anti-diabetic drug metformin decreases the odds of reporting chronic back pain in people who engage in lower levels of physical activity, are obese and are 60 years of age or older, compared to their peers not using metformin. When stratified by gender, females taking metformin who reported lower levels of physical activity or were older than 60 years of age also had lower odds of reporting chronic back pain than those females not taking metformin. Hence, clinicians should be aware that these subgroups may benefit the most from the potential positive effect that metformin has on reducing the prevalence of chronic back pain. However, the reasons why metformin reduced the odds of chronic back pain in obese, older, or less physically active people and females, in indirect comparison to the other subgroups of BMI (normal or overweight), age (<60 years) or physical activity level (moderate or high), remain unclear.

Only a few studies have previously examined the effect of metformin on musculoskeletal pain severity and prevalence. These studies produced conflicting results. [9, 13, 29, 30] For instance, no association was established between metformin use and the incidence of osteoarthritis, [29] or bodily neuropathic pain. [30] In contrast, in a large cohort of people with type 2 diabetes [12], we demonstrated that metformin is associated with reduced odds of chronic back pain [OR = 0.87;95%CI 0.81–0.93]. Metformin was also shown to be associated with less radicular pain when compared to people not using the drug [mean of -1.85 points (0–10 pain scale);95%CI -3.6 to -0.08]. [9] Our results suggest that the contradictory results may be explained by our large sample size and our adjustment of potential confounders and the investigation of specific patient phenotypes, specifically regarding BMI, age, and physical activity engagement.

Metformin is as an anti-diabetic drug which targets AMPK activation, suppresses hepatic glucose production, and promotes insulin sensitivity and glucose uptake by muscles [31]. However, the activation of AMPK also inhibits mTOR, and as a consequence influences pain [11, 31] by modulating inflammatory cytokines (e.g. tumour necrosis factor-alpha, interleukin-1B), diminishing mechanical allodynia (pain that is perceived when a non-painful stimulus is applied) [11, 32, 33] and reducing peripheral nociceptor excitability. [15–17]

It is therefore of clinical interest to investigate whether people with type 2 diabetes who take metformin report less pain than those not taking metformin. Interestingly, previous research has demonstrated that those who are female, obese, older, or sedentary have reduced AMPK activity [34], which may explain our significant findings. We speculate that among these subgroups with (assumed) lower AMPK activity, activation of AMPK via metformin may have a relatively greater effect on pain than in subgroups with higher AMPK activity. It is also possible that some of these subgroups, e.g., those who are obese or those who are older who may have had diabetes for a longer period, may have been prescribed higher doses of metformin. AMPK activation exerts powerful anti-inflammatory effects via numerous metabolic pathways, [35, 36] and AMPK activation and suppression of mTOR seem to be dose dependent. [37] Hence, a higher dosage will have more potential to activate AMPK, and thus metformin’s pleiotropic effects (including that on pain) may be more pronounced.

People with obesity, those who are older or who undertake a lower level of physical activity are reported to have lower levels of AMPK activity. For instance, AMPK activity dysregulation is evident in obesity associated with insulin resistance. In adipose tissue of people with morbid obesity who were insulin-resistant (with half of this cohort also having diabetes), AMPK activity was lower than in those who were BMI-matched but insulin-sensitive (i.e., without diabetes) [21]. All participants in our study were likely to have had lower AMPK activity because they were insulin-resistant (i.e., they had type 2 diabetes). It is likely that those in the present study who had both type 2 diabetes and obesity (compared to those with normal or overweight BMI with type 2 diabetes) were taking higher doses of metformin [38]. It is possible that those with lower AMPK activity (due to obesity and type 2 diabetes) experienced a more marked effect of metformin compared to those who were not obese (with type 2 diabetes), and/or that those who were obese were receiving high doses of metformin, thus magnifying its effects. This remains to be investigated.

Aging is another factor that influences AMPK activity, but very few studies have provided human data. One study of 91 twin pairs without diabetes demonstrated that older twins (58–66 years) had 47.7% lower vastus lateralis AMPK-γ3 activity (a regulatory subunit of AMPK) than younger twins (25–32 years), indicating that AMPK activity at rest is reduced with age. [22] In our cohort, it is possible that older people had experienced diabetes for a longer duration (with poorer pancreatic function) and hence may have been prescribed higher doses of metformin, increasing the anti-inflammatory effect and reducing chronic pain.

Furthermore, there are no studies that have investigated the combined effect of metformin and physical activity level (or exercise) on pain. However, it has been reported that AMPK activity may be dependent on exercise intensity, e.g., cycling for 20 minutes at moderate or high intensity increased the activity of a catalytic subunit of AMPK (α2) by five-to-eight-fold compared to low-intensity cycling [39]. Whilst our study did not have data available on pain specifically experienced during exercise, we included level of physical engagement and believe it is plausible that people with low levels of physical activity may have had lower AMKP activity and may have been prescribed higher doses of metformin to better control their glucose level.

We found that females who engaged in low levels of physical activity and were taking metformin had reduced odds of chronic back pain compared to those not taking metformin, whereas this relationship was not evident among men. Females have a lower expression of regulatory AMPK-subunits in skeletal muscle than men and lower AMPK activity, especially during exercise [22]. Hence, the addition of metformin in the presence of lower AMPK may have a relatively greater benefit with respect to the pain-modulating effects of metformin in females.

When we performed the multiplicative interaction between metformin and the modifiers (physical activity, BMI and Age), no multiplicative effect was observed. The protective effect of metformin for the prevalence of low back pain is more pronounced in women with high BMI and older, but the relationship is not interactive.

Another related aspect that could impact the modifier’s factors (BMI, age and physical activity) and its subgroups is the socioeconomic factors. Low socioeconomic status isa risk factor for obesity [40], and people with higher socioeconomic status are more physically active than people with lower socioeconomics status [41]. We have adjusted our analyses for the educational attainment to control for socioeconomic factors. The effects of socioeconomics on the modifier variables were beyond the scope of this study. However, we believe that future studies could investigate the effect of socioeconomic in the relationship between metformin and chronic back pain and its modifier.

Our study has several strengths. This is the first study to investigate whether physical activity, BMI, and age modify the relationship between metformin and chronic back pain in people with type 2 diabetes. Secondly, the very large sample size allowed for the stratification of the cohort into people taking or not taking metformin, as well as for large subgroups of people based on age, sex, and lifestyle. Thirdly, participants were included only if their chronic back pain was severe enough to limit their usual activities. However, this study also has some limitations that should be acknowledged. We acknowledge that self-reported questionnaires may be subject to recall bias. Even so, the questionnaire first asked if the participant had back pain in the last month, which should not be difficult for participants to recall. Participants were then asked if this pain lasted for more than three months. This may be more subject to recall bias, but given it was addressing fairly recent back pain, we feel reasonably confident that recall would reflect experiences.

Metformin dosage was not available, and it is likely that some of these subgroups (obese, older, less active) were taking higher dosages of metformin in order to achieve adequate glycaemic control. The reasons for potentially higher dosages may include that diabetes may have been present for a longer duration with the deterioration of pancreatic function (especially amongst older people) or may have been in poorer control (particularly for those with inadequate physical activity or obesity) in some of the subgroups. [42] This is important given that metformin acts on AMPK in a dose-dependent manner. [37] Cross-sectional nature of the study limits the investigation of causation, and therefore no inference can be formed on preventative measures for chronic back pain. Longitudinal data are necessary to investigate the relationship between metformin and the incidence of chronic back pain in people with type 2 diabetes.

## Conclusion

People with type 2 diabetes who were using metformin and who were obese, engaged in low levels of physical activity, or older (≥60 years) had lower odds of reporting chronic back pain than those not using metformin. Thus metformin might reduce prevalence of low back pain in this vulnerable population, however, these findings should be confirmed in studies using a longitudinal design.
